# Assessing the Performance of Food Safety Management System Using Food Safety Management System Diagnostic Tools and Microbial Assessment Scheme: A Case of Powdered Beverage Manufacturers

**DOI:** 10.21315/mjms2021.28.3.12

**Published:** 2021-06-30

**Authors:** Hua Yen Cheah, Suhaila Emma Merican, Mahmud@Ab Rashid Nor Khaizura, Ainul Zakiah Abu Bakar, Syaliza Omar, Maimunah Sanny

**Affiliations:** 1 Department of Food Science, Faculty of Food Science and Technology, Universiti Putra Malaysia, Serdang, Selangor, Malaysia; 2 Mindsky Enterprise, Kajang, Selangor, Malaysia; 3 Department of Food Service Management, Faculty of Food Science and Technology, Universiti Putra Malaysia, Serdang, Selangor, Malaysia; 4 Faculty of Pharmacy, Universiti Sultan Zainal Abidin, Besut, Terengganu, Malaysia; 5 Laboratory of Food Safety and Food Integrity, Institute of Tropical Agricultural and Food Security, Universiti Putra Malaysia, Serdang, Selangor, Malaysia

**Keywords:** FSMS-DI, MAS, MeSTI (Food Safety is the Responsibility of the Industry), good manufacturing practices, hazard analysis critical control point, ISO 22000 Food Safety Management System, powdered beverages

## Abstract

**Background:**

The objective of the study is to assess the performance of the Food Safety Management System (FSMS) among powdered beverage manufacturers using Food Safety Management System Diagnostic Tools (FSMS-DI) and Microbial Assessment Scheme (MAS).

**Methods:**

FSMS-DI was used to evaluate the context factors, core control and core assurance activities of five powdered beverage manufacturers with different types of FSMS certification. Manufacturer A is not certified with any FSMS, while manufacturers B, C, D and E are complied with MeSTI, GMP, HACCP and ISO 22000, respectively. For MAS, samples were collected from the selected critical sampling locations of two manufacturers who complied FSMS with the least (manufacturer B) and the most stringent (manufacturer E) requirements. The samples consisted of two different types of powdered beverage products were analysed for total plate count (TPC), *Salmonella, Escherichia coli*, *Staphylococcus aureus*, yeast and mould count (YMC). *Results*: The food safety (FS) output of powdered beverages for manufacturer E was better (overall score of 3) than manufacturer B (overall score of 2–3). Manufacturer E was able to achieve their FS objectives. The FSMS activities of manufacturer C, D and E were better (overall score of 2–3) than manufacturer A and B (overall score of 1–2).

**Conclusion:**

The study demonstrated that FSMS-DI and MAS can be used to differentiate the FSMS performance of powdered beverage manufacturers with different types of FSMS certification. Higher scores of FSMS activities obtained by the manufacturer who complied with stringent FSMS certifications contributed to better microbiological safety performance of powdered beverages.

## Introduction

The powdered beverage is a low-risk product formulated with powdered ingredients that are low in moisture content. It is also a high care product with minimum process control, manual processing and without heat treatment at the manufacturer but requires minimum preparation at the point of consumption by the consumer (1). The powdered beverage is a mixture of different ingredients that in all cases have different characteristics and specifications (2). It can be made not only using whey protein powder but also using a multitude of fruits, vegetables, herbs and other ingredients (3). The powdered beverage can be contaminated by the food handlers with a wide variety of microorganisms during manufacturing and packaging processes (4). Recent reports have shown that powdered beverages are good culture media for bacteria and fungi despite their low moisture content and powdery nature (5).

Regulations forced food business operators to implement a Food Safety Management System (FSMS) in order to assure the safety of food products (6, 7). In Malaysia, the Food Hygiene Regulations 2009 demands all food manufacturers to provide and make available a FSMS based on any of the following standards: Good Manufacturing Practices (GMP), Hazard Analysis Critical Control Points (HACCP) principles or ISO 22000 FSMS (8, 9). The ISO 22000 was introduced in 2005 to bridge the gap between ISO 9001:2000 and HACCP. The ISO 22000 series integrate the principles of the HACCP system with prerequisite programmes, such as GMP and good hygiene practices (GHP), thus ensures that there are no weak links in the food supply chain (10). In addition, *Makanan Selamat Tanggungjawab Industri* (MeSTI) or the ‘Food Safety is the Responsibility of the Industry’, is a nationally recognised system introduced by the Ministry of Health Malaysia to put in place a system for the maintenance of food hygiene and process control, which includes food safety (FS) assurance and food traceability. All food manufacturing establishments in Malaysia that do not comply with the GMP, HACCP or ISO 22000-compliant are encouraged to seek MeSTI’s certification (11). There are differences in specific requirements of each standard (12). The requirements of ISO 22000 are regarded as the most stringent among the mentioned standards. Nevertheless, the requirements of FSMS can be translated differently due to the different processes and different sizes or positions of the different stakeholders in the food chain (10, 13, 14).

Stakeholder requirements force manufacturers to analyse their FSMS performance to improve FS (15). According to Luning et al. (16), the performance of FSMS in agrifood products could be evaluated using a systematic assessment tool. The FSMS diagnostic (FSMS-DI) tool aims to evaluate the performance of FSMS activities with the microbiological result (Microbial Assessment Scheme [MAS]) as output (16). The diagnostic tools provide a comprehensive checklist for the analysis of the FSMS environment (context factors), core control activities and core assurance activities. Recently, the performance of FSMS using diagnostic tools and MAS have been conducted in a lamb chain (14), food service establishments (17, 18), hot pepper and green bean farms (19), fresh produce chain (20) and meat processing industries (15). Kohilavani et al. (1) discussed the establishment of a HACCP system in selected powdered beverage manufacturers but focused mainly on the development of the system. To the best of the author’s knowledge, FSMS performance of powdered beverage manufacturers with different types of FSMS certification has not been assessed yet. Hence, this study was conducted to assess the FSMS performance of powdered beverage manufacturers using FSMS-DI and MAS. Five powdered beverage manufacturers with different types of FSMS certification, i.e. MeSTI, GMP, HACCP and ISO 22000 FSMS (including one manufacturer with no FSMS certification) were assessed. Their leaders of the FS Team were involved in the FSMS-DI. Questionnaire elements were context factors, core control and core assurance activities. The FSMS assessment results were verified with microbiological results as output.

## Methods

### Characteristic of Manufacturers

Five small and medium enterprises (SMEs) with between 10 and 20 employees, who manufactured powdered beverage products, were involved in this study. They were selected based on the types of FSMS certification that they obtained and their willingness to participate in the study. Manufacturer A is not certified with any FSMS, manufacturer B is certified with MeSTI, manufacturer C is certified with GMP, manufacturer D is certified with HACCP and manufacturer E is certified with ISO 22000 FSMS. The manufacturers produced two different types of powdered beverage products, i.e. animal- and plant-based. Animal-based powdered beverages used either dairy or nondairy creamer in the formulation whereas plant-based powdered beverages used botanical ingredients such as ginseng powder, tongkat ali powder and rose petal powder; with neither dairy nor non-dairy creamer was added in the formulation.

### Food Safety Management System Diagnosis Tool

FSMS-DI tool adapted from Osés et al. (14) was employed to obtain insight into the performance of FSMS in all five manufacturers. The FSMS-DI consists of three sections. Each section has a set of indicators and grids with a brief description of the indicator. Levels 1, 2 and 3 represent the low, moderate and high risk. Section I is the context factors assessment for the characteristics of the product, process/production, organisational and chain environment. Section II is a diagnosis of the core control activities in a company, which include design preventive measures, design intervention processes, design monitoring system and actual operation control strategies. Section III is a diagnosis of the core assurance activities such as defining documentation system, validation and verification.

An in-depth interview was conducted and the FS Team leaders of all five manufacturers were asked to choose a grid for each of the indicators that represents their FSMS situation. FS Team leader must hold the role of either Operation Director or Quality Control Manager or Production Manager. The interview process lasted 2 h–3 h and was followed by an on-site visit to confirm the assessment.

Total scores for all sections were calculated by total up all the indicators grid in each of the section and divided by the total number of indicators in each section. The average score for each section was interpreted according to the method described by Osés et al. (14).

### Food Safety Output Diagnosis

MAS as described by Jacxsens et al. (6), was used to obtain insight into the FS output of powdered beverage manufacturers. Both animal-and plant-based of powdered beverage samples were collected from the manufacturer who complied FSMS with the least (manufacturer B) and the most stringent (manufacturer E) requirements. No samples were collected from manufacturers A, C and D.

### Selected Critical Sampling Locations

Critical sampling locations (CSLs) are the processing locations with high a risk of contamination, in which microbial might survive and contaminate the next processing (6). The following sampling points were selected: final products (animal- and plant-based of powdered beverages), production equipment (inner surface of mixer, inner surface of filling funnel, inner surface of container, and scoop), personal hygiene (before and after washing hand), tap water and air quality.

### Sampling Frequencies

A visit was arranged to each powdered beverage manufacturer and samples were collected at all the selected CSLs. Two samples were taken each for the final products (animal-and plant-based of powdered beverages), tap water, air quality, food contact surfaces and food handlers’ hands (before and after hand washing). A total of 88 samples (11 CSLs × 2 manufacturers × 2 types of powdered beverages × 2 replicates) were collected.

### Selection of Microbiological Parameters and Analytical Methods

The selection of microbiological parameters was done according to the Guidelines for the Microbiological Examination of Ready-to-Eat Foods (21). *Salmonella* and *Bacillus cereus* were selected as FS indicators. *Staphylococcus aureus* and *Escherichia coli* were selected as the hygiene indicators. Total plate count (TPC) was selected as the total microbiological quality indicator. Yeast and mould count (YMC) was selected as the environmental quality indicator. A sample size of powdered beverage products for microbiological analysis was taken according to Pomeranz and Meloan (22).

### Sample Preparation for Microbiological Analysis of Final Products

The enumeration of TPC, *Escherichia coli* and *Staphylococcus aureus* counts and detection of *Salmonella* and *Bacillus cereus* were performed according to ICMSF (23). Twenty-five grams of the 300 g of food sample was homogenised with 225 mL of buffered peptone water for 2 min in a sterile stomacher bag using a stomacher. Homogenised samples were then serially diluted with 1% sterile peptone water up to 10^−4^ dilutions. Then, 0.1 mL of the samples at each dilution factor was transferred onto their selective agars. Plate count agar (PCA; Oxoid, UK) was used for TPC, eosin methylene blue agar (EMB; Oxoid, UK) was used for *Escherichia coli*, and Baird Packer Agar (BP Agar; Oxoid, UK) was used for *Staphylococcus aureus*. The detection of *Salmonella* and *Bacillus cereus* was performed using xylose lysine deoxycholate agar (XLD; Oxoid, UK) and *Bacillus cereus* selective agar base (BCSA; Oxoid, UK), respectively. All medium plates were incubated at 37 °C for 24 h–48 h, except the PDA plates which were incubated at 37 °C for 5 days. Only 25–250 colonies on the plates were counted using a colony counter (Today’s Instruments, Taiwan). Results were expressed as colony forming units per gram (CFU/g). Isolates microbial were identified by cultural, morphological and physiological characteristics as described in media supplier (Oxoid, UK).

### Air Quality Sampling

The air quality of production areas was inspected using culture settling plate technique according to Salustiano et al. (24). Potato dextrose agar (PDA) plates were opened and exposed at the processing area for 15 min. The petri dishes were closed and incubated at 35 °C–37 °C for 5 days. Only 25–250 colony forming unit (CFU) were counted on plates using a colony counter (Today’s Instruments, Taiwan) and expressed as CFU per cubic meter (CFU/m^3^).

### Personnel Hygienic with Hand Swab

Sampling was done before and after the production staff washed their hand in changing room according to ISO 18593 (25). Swabbing was obtained by using sterile cotton swab wet with 1% peptone water (sample 10^−1^). Then, a sterilised sampling stick was swabbed on 25 cm^2^ hand palm. After swabbing, the swab head was gently immersed in 1% of the same peptone water. The sterilised peptone water 1% was later kept in ice box. Samples were sent to the laboratory for analysis within 3 h to detect *Staphylococcus aureus*. Baird Parker (BP) agar plate was used to sample *Staphylococcus aureus* on hand. Sample 10^−1^ was serially diluted up to 10^−3^ dilution. Next, 0.1 mL of peptone water was pipetted and transferred on the surface of BP agar. The culture BP agar plates were incubated at 37 °C for 24 h–48 h. Following incubation, 15–150 colonies were counted using a colony counter (Today’s Instruments, Taiwan) and the results were expressed in CFU/cm^2^. Isolates obtained were identified by cultural, morphological and physiological characteristics as described in media supplier (Oxoid, UK).

### Sampling for Food Contact Surface

Sampling of the food contact surfaces after the machines and equipment were cleaned was also conducted as described in Personnel Hygienic with Hand Swab section above. Eosin methylene blue (EMB) agar plate was used to sample *Escherichia coli*, meanwhile, PDA was used to sample yeast and mould. The culture EMB agar plates were incubated at 37 °C for 24 h–48 h. PDA was cultured at 37 °C for 5 days.

### Sampling of Tap Water

*Escherichia coli* counts of tap water samples were performed according to Nik Rosmawati et al. (26). Serial dilutions up to 10^−6^ were prepared, and 0.1 mL of the samples at each dilution factor was transferred onto EMB agar plates and spread evenly. Inoculated EMB agar plates were incubated at 37 °C for 18 h–24 h. Only 25–250 colonies on the plates were counted using a colony counter (Today’s Instruments, Taiwan) and the results were expressed as CFU/mL. The isolates obtained were identified as described by the media supplier (Oxoid, UK).

### Data Processing and Interpretation

The enumerated count from raw material, final products, hand and food contact surfaces were compared against the Guidelines for the Microbiological Examination of Ready-to-Eat Foods (21) and considered unsafe for consumption if the enumerated count was higher than the permitted level. According to FSANZ (21), the permitted levels for the microbial contamination are *Escherichia coli* < 3 CFU/g, *Staphylococcus aureus* < 10^2^ CFU/g, *Salmonella* must be absent in 25 g, *Bacillus cereus* < 10^2^ CFU/g and TPC < 10^4^ CFU/g. The microbial counts of hands and food contact surfaces were considered unacceptable when the microbial contamination is equal to or higher than that present in the food samples (27). *Escherichia coli* in tap water must be absent in 100 mL according to Malaysia Food Act and Regulations (28). The maximum value for YMC must not exceed 90 CFU/m^3^ as recommended by the American Public Health Association (29).

Microbiological results from all the CSLs were classified from 1–3 to indicate the microbiological safety level according to Jacxsens et al. (6). Level 3 represents good FS performance (legal criteria or guidelines are respected, no improvements are needed), level 2 represents a moderate FS performance (legal criteria or guideline are exceeded but can be attributed to a specific control activity in the FSMS) and level 1 represents a poor FS performance (legal criteria or guideline are exceeded and can be attributed to several control activities). The sum of the levels of the individual microbiological parameters analysed is reflected in a FS level profile. The sum of the FS levels for each final product in this study might reach a maximum of 18 (6 × 3). An overall score of 1 (poor risk) was assigned when the sum of the levels was 6–7, scores of 1–2 (poor to moderate level) when the sum of the levels was 8–10, scores of 2 (moderate-risk) when the sum of the levels was 11–13, scores of 2–3 (moderate to good level) when the sum of the levels was 14–16 and a score of 3 (good level) when the sum of the levels was 17–18.

## Results

Table 1 shows that all five manufacturers have a moderate risk (score 2) for their context factors, irrespective of the types of FSMS they certified to. Manufacturers A and B obtained a basic to average level (overall score of 1–2) of FSMS. Manufacturers D and E obtained a moderate to a high level (overall score of 2–3) of FSMS. Table 2 shows that the overall score of FS output of animal- and plant-based powdered beverages for manufacturer B (MeSTI certified) was ‘moderate to good level’ (overall score of 2–3), whereas for manufacturer E (ISO 22000 FSMS certified) was ‘good level’ (overall score of 3). One FS indicator (i.e. *Salmonella*), one hygiene indicator (i.e. *Escherichia coli*) and air quality (YMC) received a good FS level of 3 for all manufacturers, as legal criteria or guidelines were respected at all corresponding CSLs (Table 2). However, the other hygiene indicator (i.e. *Staphylococcus aureus*) received a medium FS level of 2 for all manufacturers. Furthermore, the total microbiological quality indicator (TPC) and another FS indicator (i.e. *Bacillus cereus*) for manufacturer E was better (overall score of 3) than manufacturer B (overall score of 2 for plant-based powdered beverages, although animal-based powdered beverages scored a good FS level of 3).

## Discussion

The present work assessed the performance of powdered beverage manufacturers with different types of FSMS certification using FSMS-DI and MAS. It was found that the FS output of animal- and plant-based powdered beverages for manufacturer E (ISO 22000 FSMS certified) scored a ‘good level’ (overall score of 3) whereas for manufacturer B (MeSTI certified) only scored a ‘moderate to good level’ (overall score of 2–3). The microbiological parameters selected in the MAS were the manufacturers’ FS objectives. Manufacturer E was able to achieve their FS objectives, i.e. a good level of FS performance, for both animal- and plant-based powdered beverages. In addition, TPC was not detected in powdered beverages of manufacturer E but their levels were above the permitted limit in samples from manufacturer B. It appeared that the microbiological safety performance of the manufacturer who complied with stringent FSMS was better than the manufacturer who complied with less stringent FSMS. The findings were consistent with the results of Nyarugwe et al. (30) as well as De Boeck et al. (31) who reported that companies with well-established FSMS had a better microbiological safety performance.

In addition, *Bacillus cereus* was detected in plant-based powdered beverages at levels that exceeded the limit for manufacturer B but not in animal-based powdered beverages. Furthermore, *Bacillus cereus* was not detected in both animal-and plant-based powdered beverages produced by manufacturer E. The presence of *Bacillus cereus* in plant-based powdered beverages above the permitted limits in manufacturer B (which contributed to the low scores obtained) was in agreement with Little et al. (32) who also reported that the ready-to-eat foods added with botanical ingredients in the form of spices were contaminated with *Bacillus cereus* above the acceptable limit. *Bacillus cereus* spores survive in dry foods and dry food processing environments such as powdered beverages if they are not properly processed or stored (33). Further, it appeared that *Bacillus cereus* contaminated plant-based powdered beverages above the acceptable limit for manufacturer B who complied with the least stringent FSMS requirements. As mentioned, plant-based powdered beverages used botanical ingredients such as ginseng powder, tongkat ali powder and rose petal powder in the formulation and these ingredients are typically not sterile. Due to their origin, plant materials are frequently subject to contamination by microorganisms such as Bacillus cereus from the soil, air and water (34). Other studies suggested that poor sanitation, cross-contamination, improper maintenance, poor equipment and facility design, and lack of proper HACCP and GMP can contribute to the contamination of botanical ingredients (35, 36). The present study showed manufacturer B who complied with the least stringent FSMS requirements (MeSTI) could not control their microbiological FS output, especially on Bacillus cereus counts in contrast to manufacturer E who complied with the most stringent FSMS (ISO 22000 FSMS). MeSTI is a pre-certification scheme devised by the Ministry of Health Malaysia for local small manufacturers to serve as an entry point into FS certification. The local small manufacturers are advised to adopt HACCP/GMP certification in two years after the pre-certification scheme (37).

Moreover, *Staphylococcus aureus* was not detected after hand washing although their levels were exceeded the limit before hand washing for both manufacturers. The results were consistent with Matuka et al. (38) who monitored *Staphylococcus aureus* levels on the hands (before and after handwashing) of theatre staff in three hospitals in Johannesburg, South Africa. They reported that almost half of the theatre staff carried *Staphylococcus aureus* isolates on their hands prior to handwashing. Although *Salmonella* is the principal food pathogen associated with botanical ingredients (39), it was not detected in animal- and plant-based powdered beverages for both manufacturers in the present study. The results were consistent with the report released by the Centre for Food Safety (40) who monitored the microbiological quality of some cold-served powdered beverages in Hong Kong and reported that *Salmonella* was not detected in all 198 samples collected.

In addition, all five manufacturers have a moderate risk (score 2) for their context factors, irrespective of the types of FSMS they certified to. A context factor is defined as structural elements of a situation that affect decision-making activities in the FSMS and its FS output (41). All five manufacturers who participated in the study were in the same nature of business, therefore they were having similar risk (at a moderate level) of context factors: product, process, organisational and production chain environment. More in details about moderate risk is that there are potential chances of pathogen and microorganisms contamination on the process characteristics and the powdered beverage products. The results were consistent with Osés et al. (14) who reported the overall context scores was 2 (moderate-risk) for all actors, i.e. slaughtering house, retail shop, and processing plant, along the lamb chain. Kussaga et al. (42) also reported the overall context score was 2 (moderate-risk) in a Nile perch processing company in Tanzania.

FSMS implemented in a food processing industry is based on GHP, HACCP principles, and should address both FS control and assurance activities to guarantee FS (6). The FSMS activities diagnostic tool that was used in the present study was developed based on the assumption that a higher/more sophisticated level of control and assurance activities means that the food manufacturer has a more advanced FS management system in place, and can control their microbiological FS output better (7, 16, 43). The results in the present study revealed that manufacturers who complied with the stringent FSMS obtained a moderate to a high level (overall score of 2–3) for their core control and assurance activities. In contrast, manufacturers who complied with the less stringent FSMS obtained a basic to average level (overall score of 1–2) for their core control and assurance activities. As supported by Luning et al. (15), manufacturers that are operating in a moderate-risk context require average level of core control and assurance activities to realise a good FS output. The present study showed that manufacturer E, which has an FSMS at a moderate to a high level (overall score of 2–3) achieved their FS objectives, i.e. a good level of FS performance. However, manufacturer B, which has an FSMS at a basic to average level (overall score of 1–2) scored ‘moderate to good level’ (overall score of 2–3) of FS output, therefore unable to achieve their FS objectives. It is apparent that MeSTI certification is insufficient to put in place a higher/more sophisticated level of control and assurance activities to realise a good FS output. This finding was consistent with Bilska and Kołożyn-Krajewska (44) who suggested that lack of monitoring of raw materials and operations could increase the FS risk of final products. Powdered beverage manufacturers with MeSTI certification should be encouraged to adopt a more stringent standard to assure the microbiological safety performance of powdered beverages.

## Conclusion

The present study demonstrated that FSMS-DI and MAS can be used to differentiate the FSMS performance of the powdered beverage manufacturers with different types of FSMS certification. Since powdered beverage businesses are operating in a moderate-risk context, higher scores of FSMS activities have resulted in better microbiological safety performance of powdered beverage for manufacturer implementing stringent when compared to less stringent FSMS. MeSTI certification is insufficient to implement a higher/more sophisticated level of control and assurance activities to realise a good FS output. The powdered beverage manufacturers with MeSTI certification are encouraged to adopt a more stringent standard to assure the microbiological safety performance of their products.

## Figures and Tables

**Table 1:** Scores attributed to the indicators representing the context factors, core control and core assurance activities of powdered beverage manufacturers with different types management (FSMS) certification

Indicators	Manufacturers				

	A^1^	B^2^	C^3^	D^4^	E^5^
**I. Context factors (overall)**^6^	2	2	2	2	2
**Product characteristics**					
Risk of raw materials	2^7^	2	2	2	2
Risk of final product groups	3	1	3	2	2
Safety contribution packaging concept	2	2	3	2	2
Microbial risk of initial materials	2	2	1	2	2
Risk of initial materials to mycotoxins, e.g. aflatoxin	1	1	1	3	1
Microbiological risk of final product	2	1	1	1	1
**Process/Production characteristics**					
Extent intervention steps	3	1	2	3	3
Degree production process changes	3	3	1	3	3
Rate product and process design changes	2	3	3	3	2
Susceptibility of production system	2	2	2	2	1
Susceptibility of water supply	1	1	1	1	1
Susceptibility to flooding	1	1	1	1	1
Risk of production site location	1	1	1	1	1
**Organisational characteristics**					
Presence of technological staff	3	3	3	3	3
Variability workforce composition	2	3	2	2	2
Sufficiency operators’ competence	2	3	2	2	2
Extent of management commitment	3	3	2	3	2
Degree of employee involvement	2	3	2	3	2
Level of formalisation	2	3	3	2	2
Sufficiency supporting information systems	2	3	3	2	2
**Chain environmental characteristics**					
Degree safety contribution in chain position	2	3	2	2	2
Extent of power in supplier relationships	2	3	3	3	2
Degree of authority in customer relationships/sufficiency of FS authority	3	3	2	2	2
Severity of stakeholders’ requirements	1	1	1	1	1
Degree of information exchange in supply chain	3	3	2	2	2
Sophistication of logistic infrastructure	2	3	2	2	2
Supportiveness of FS authority	3	2	2	2	2
Degree of globalisation of supply	3	2	2	2	2
Specificity of external supply	2	2	3	2	2
Specificity of food safety legal framework	2	3	1	2	2
**II. FSMS activities (overall)**^8^	1–2	1–2	2	2–3	2–3
**Core control activities**					
**Design preventive measures**					
Sophistication hygienic design equipment and facilities	1^9^	1	1	2	2
Adequacy heat treatment facilities, e.g. pasteurisation, cooling	1	1	1	2	2
Specificity sanitation program	1	1	2	2	2
Extent personal hygiene requirements	2	1	1	2	2
Design intervention processes					
Adequacy physical intervention equipment methods	2	1	1	2	2
Specificity maintenance and calibration programs intervention equipment	1	2	2	3	2
Specificity and effectiveness intervention methods	1	1	1	1	1
**Design monitoring system**					
Appropriateness CCP analysis	1	1	3	3	2
Appropriateness standards and tolerances design	1	1	2	3	2
Appropriateness of limits and tolerance assessment	1	1	2	2	2
Adequacy analytical methods to assess pathogen levels	2	2	2	3	2
Adequacy of measuring and analytical equipment to monitoring process/product status	1	1	2	2	2
Specificity calibration and verification program for measuring or analytical equipment	1	2	2	3	2
Specificity sampling design (microbial assessment) and measuring plan	1	1	1	2	2
Extent corrective actions	1	1	1	1	2
Actual operation control strategies					
Actual availability of procedures for cleaning, personal hygiene, maintenance and calibration intervention equipment, calibration and verification measuring and analytical equipment, CCP control	1	1	2	1	2
Actual/Extent compliance to procedures, practices as what you did is what you write	1	1	2	2	2
Actual hygienic performance of equipment and facilities	2	2	3	2	3
Actual processing equipment performance	2	2	1	2	3
Actual process capability of physical intervention processes	2	2	3	2	2
Actual analytical/measuring equipment performance	2	2	3	3	3
Actual process capability of packaging intervention equipment	2	2	3	3	3
**III. Core assurance activities**					
Sophistication translating external requirements, e.g. stoke holder into internal FSMS requirements	1	1	2	3	3
**Validation**					
Sophistication validating preventive measures including preventive equipment and facilities, sanitation and personal hygiene programmes	2	2	2	2	2
Sophistication validating effectiveness intervention system (process, equipment and methods) similar as for preventive measures	1	1	3	2	1
Sophistication of validating monitoring systems (CCP and control points)	1	1	3	2	3
**Verification**					
Extent of verifying people related performance, e.g. procedure characteristics and procedure compliance	1	2	3	3	3
Extent of verifying equipment and methods related performance prevention and intervention equipment/method measuring/analysis equipment	1	2	3	2	3
**Documentation system**					
Appropriateness of documentation system	1	2	2	3	3
Appropriateness of record keeping system	2	2	2	3	3

Notes:

1 Company not certified with any FSMS

2 Company certified with MeSTI

3 Company certified with GMP

4 Company certified with HACCP

5 Company certified with ISO 22000 FSMS

6 In case of context factors, scores in bold are assigned overall scores [If a mean score for the context factors was between 1 and 1.2 then the assigned score is 1, if between 1.3 and 1.7 (assigned score 1–2), if between 1.8 and 2.2 (2), if between 2.3 and 2.7 (2–3), and if between 2.8 and 3.0 then assigned score 3]

7 Context scores, 1 indicates low risk, 2 moderate risk, 3 high risk;

8 In case of core control and assurance activities, scores in bold are assigned overall scores [If a mean score was between 0 and 0.2 then the assigned score is 0, if between 0.3 and 1.2 (assigned score 1), if between 1.3 and 1.7 (1–2), if between 1.8 and 2.2 (2), if between 2.3 and 2.7 (2–3), and if the mean score was between 2.8 and 3.0 then the assigned score is 3

9 Activity scores, 0 indicates low level (absence, not applied), 1 basic level, 2 average level, 3 advanced level

**Table 2:** Number of samples exceeding the limiting criteria for total plate count, *Salmonella, Escherichia coli*, *Staphylococcus aureus, Bacillus cereus* and YMC over the different critical sampling locations, the FS level attributed for all microbiological parameters, and the total FS output of manufacturers with different types of Food Safety Management (FSMS) certification, producing animal-and plant-based powdered beverage products

CSL^1^		Manufacturers			

		B^2^		E^3^	
	
		Animal-based powdered beverage products	Plant-based powdered beverage products	Animal-based powdered beverage products	Plant-based powdered beverage products
**TPC**					
Final product (*n*^4^ = 2)	1	2	2	ND^6^	ND
FS level^7^		2	2	3	3
***Salmonella***					
Final product (*n* = 2)	1	ND	ND	ND	ND
FS level		3	3	3	3
***Escherichia coli***					
Final product (*n* = 2)	1	ND	ND	ND	ND
Water quality (*n* = 2)	2	ND	ND	ND	ND
Surfaces					
Scope (*n* = 2)	3	ND	ND	ND	ND
Container (*n* = 2)	4	ND	ND	ND	ND
Mixer machine (*n* = 2)	5	ND	ND	ND	ND
Filling funnel (*n* = 2)	6	ND	ND	ND	ND
FS level		3	3	3	3
***Staphylococcus aureus***					
Final product (*n* = 2)	1	ND	ND	ND	ND
Personnel hygiene:					
Before hand washing (*n* = 2)	2	2	2	2	2
After hand washing (*n* = 2)	3	ND	ND	ND	ND
FS level		2	2	2	2
* Bacillus cereus*					
Final product (*n* = 2)	1	ND	2	ND	ND
FS level		3	2	3	3
**YMC**					
Air quality :					
Grinding room (*n* = 2)	1	0^5^	0	ND	ND
Filling room (*n* = 2) 2	ND	ND	ND	ND	
Mixing room (*n* = 2)	3	ND	ND	ND	ND
FS level		3	3	3	3
**FS output**^8^		**2**–**3**	**2**–**3**	3	3

Notes:

1 CSL = critical sampling location

2 Company certified with MeSTI

3 Company certified with ISO 22000 FSMS

4 Total number of samples per CSL

5 Counts were found but below criteria

6 Below detection limit (ND)

7 FS level is classified from 1 to 3, where level 1 is a low result (legal criteria or guidelines are exceeded, improvements need to be made on multiple control activities of the FSMS), level 2 is a medium result (legal criteria or guidelines are exceeded, improvements need to be made on a single control activity of the FSMS) and level 3 is a good result (legal criteria or guidelines are respected, no improvements needed — current level of FSMS is high enough to cover the hazard)

8 An overall score of 1 (poor risk) was assigned when the sum of the levels was 6–7, scores of 1–2 (poor to moderate level) when the sum of the levels was 8–10, scores of 2 (moderate-risk) when the sum of the levels was 11–13, scores of 2–3 (moderate to good level) when the sum of the levels was 14–16, and a score of 3 (good level) when the sum of the levels was 17–18
